# Targeted therapies in atopic dermatitis: toward endotype-driven precision medicine and disease modification

**DOI:** 10.3389/fimmu.2026.1959035

**Published:** 2026-10-07

**Authors:** Wei Song, Xiaorui Yao, Changzhen Lei, Shiya Yao

**Affiliations:** 1 Department of Plastic and Aesthetic Surgery, Lishui Traditional Chinese Medicine Hospital, Lishui, Zhejiang, China; 2 Lishui Central Hospital, Shaoxing University, Lishui, Zhejiang, China; 3 Department of Gastrointestinal Surgery, Shanghai General Hospital, Shanghai Jiao Tong University School of Medicine, Shanghai, China; 4 Gongji Gastrointestinal Tumor Research Institute, Shanghai General Hospital, Shanghai Jiao Tong University School of Medicine, Shanghai, China

**Keywords:** atopic dermatitis, biologics, disease modification, JAK inhibitors, precision medicine, targeted therapy

## Abstract

Atopic dermatitis (AD) is a chronic, relapsing inflammatory skin disease driven by a combination of genetic predisposition, epidermal barrier dysfunction, immune dysregulation, aberrant neuroinflammatory signaling, and environmental factors. Genetic defects in barrier-related molecules, particularly loss-of-function mutations in filaggrin (FLG), represent key heritable determinants of impaired epidermal integrity and enhanced allergen penetration. In recent years, advances in the understanding of its molecular pathogenesis have progressively shifted the therapeutic paradigm from conventional broad-spectrum immunosuppression toward precision-targeted interventions aimed at key inflammatory pathways, substantially transforming disease management in patients with moderate-to-severe AD. Current evidence indicates that type 2 immune inflammation remains a central pathogenic axis in AD. Key mediators, including interleukin (IL)-4, IL-13, IL-31, thymic stromal lymphopoietin (TSLP), and the Janus kinase (JAK)–signal transducer and activator of transcription (STAT) signaling pathway, collectively contribute to epidermal barrier disruption, inflammatory amplification, and the itch–scratch cycle. Based on these mechanisms, a range of biologics and small-molecule targeted therapies—including IL-4 receptor α (IL-4Rα) antagonists, IL-13 monoclonal antibodies, IL-31 pathway inhibitors, JAK inhibitors, and emerging OX40/OX40L modulatory strategies—have demonstrated significant efficacy in randomized controlled trials and real-world studies, thereby ushering AD into a mechanism-driven therapeutic era. However, targeted therapies still face critical challenges, including heterogeneous treatment responses, insufficient long-term safety data, limited stratification of disease endotypes, and disparities in treatment accessibility. Increasing evidence suggests that therapeutic response is more closely associated with immunological endotypes than traditional clinical phenotypes, and that single-pathway blockade alone is insufficient to achieve durable disease modification. This review systematically summarizes the key pathogenic mechanisms of AD, current targeted therapeutic strategies, and their clinical translation progress, with a particular focus on existing therapeutic challenges and future directions. Ultimately, integration of biomarkers, multi-omics profiling, real-world evidence, and artificial intelligence–assisted decision-making may facilitate the development of endotype-based precision treatment frameworks, enabling a shift from disease control toward sustained remission and true disease modification.

## 1 Introduction

Atopic dermatitis (AD) is a common chronic inflammatory skin disease characterized by recurrent eczematous lesions, intense pruritus, and a substantial impairment in quality of life (1). AD affects up to 20% of children and 10% of adults worldwide (2–4), imposing a considerable psychosocial and socioeconomic burden, and is frequently associated with other atopic comorbidities, including asthma, allergic rhinitis, food allergies, and other atopic disorders (5, 6).

Traditionally, AD has been regarded primarily as a type 2 (T2)-driven disease —defined as an inflammatory response orchestrated by T helper 2 (Th2) cells, type 2 innate lymphoid cells (ILC2s), and signature cytokines including interleukin (IL)-4, IL-5, and IL-13, which collectively promote eosinophilic infiltration, IgE production, and allergic inflammation (7)—resulting from the interplay among epidermal barrier dysfunction, immune dysregulation, and environmental triggers (8, 9). This type 2 pathway is distinct from type 1 (Th1/IFN-γ-mediated) and type 3 (Th17/IL-17-mediated) immune responses, which may also contribute to AD pathogenesis in specific patient subsets (7). However, accumulating evidence suggests that the immunopathology of AD extends far beyond classical T2 inflammation (10–12). Dysregulated activation of Th1, Th17, and Th22 pathways, neuroimmune interactions, microbial dysbiosis, and tissue-resident immune memory collectively contribute to the chronicity and relapse of the disease (13–15). These findings have substantially reshaped our understanding of AD, redefining it as a biologically heterogeneous disorder composed of multiple immune endotypes rather than a single disease entity (16, 17). The conceptual framework of “endotype-driven precision therapy” is grounded in recent landmark studies demonstrating that AD comprises distinct immunological subtypes—such as type 2-dominant versus mixed Th1/Th17/Th22 endotypes—which exhibit differential responses to targeted interventions (18). For instance, unbiased tissue transcriptome analyses have identified six distinct skin phenotypes linked to specific endotypes, with type 2 and type 17 responses associated with major skin phenotypes such as erythema and induration (7).

Advances in the understanding of the immunopathogenesis of AD have profoundly transformed therapeutic strategies. Over the past decade, multiple targeted therapies—including biologics directed against IL-4/IL-13 and IL-31 signaling pathways, as well as small-molecule Janus kinase (JAK) inhibitors—have substantially improved disease control in patients with moderate-to-severe AD (19, 20) (21). However, significant challenges remain. A considerable proportion of patients exhibit incomplete responses, experience disease relapse after treatment discontinuation, or fail to achieve sustained remission, highlighting the limitations of a one-size-fits-all therapeutic paradigm.

Therefore, the therapeutic goals of AD are shifting from short-term symptom control toward precision medicine and disease modification. The identification of immune endotypes, discovery of predictive biomarkers, integration of multi-omics technologies, and development of artificial intelligence–assisted clinical decision-making systems are expected to facilitate the implementation of personalized treatment strategies and ultimately reshape the natural history of the disease.

In this review, we summarize the latest advances in the immunopathogenesis of AD and critically discuss current and emerging targeted therapies from the perspective of endotype-driven precision medicine and disease modification. We further highlight the major challenges and future directions that may enable a transition from reactive disease management to predictive and personalized immunological intervention.

## 2 Integrated immunopathogenesis of atopic dermatitis

AD is a highly heterogeneous inflammatory skin disease arising from a complex interplay among epidermal barrier defects, immune dysregulation, neuroimmune interactions, microbial perturbations, and environmental exposures (22). AD is no longer considered a disease driven by a single cytokine axis, but is increasingly recognized as a dynamic immunological spectrum composed of multiple pathogenic phenotypes. These interconnected biological processes collectively determine disease initiation, persistence, therapeutic responsiveness, and propensity for relapse. A comprehensive understanding of this integrative pathogenic network provides a mechanistic foundation for the development of precision therapeutics and disease-modifying strategies.

### 2.1 Epidermal barrier dysfunction: the initiating event

Epidermal barrier dysfunction is increasingly recognized as a primary initiating event in the pathogenesis of AD, rather than merely a secondary consequence of inflammation (23). The integrity of the epidermal barrier depends on the coordinated interaction among structural proteins, intercellular lipids, and antimicrobial defense systems (24). Genetic defects in barrier-related molecules, particularly loss-of-function mutations in filaggrin (FLG), lead to impaired keratinocyte differentiation, increased transepidermal water loss, and enhanced penetration of environmental allergens and microbial products (23, 25, 26).

Barrier disruption also induces the release of epithelial-derived alarmins, including thymic stromal lymphopoietin (TSLP), IL-25, and IL-33 (27–29). These mediators activate dendritic cells and type 2 innate lymphoid cells (ILC2s), thereby promoting type 2 immune polarization and establishing a pro-inflammatory microenvironment (30–32). In addition, impaired barrier integrity is closely associated with microbial dysbiosis, particularly *Staphylococcus aureus* colonization, which further amplifies cutaneous inflammation and exacerbates barrier damage (23, 25, 26).

Importantly, epidermal barrier dysfunction and immune activation form a self-perpetuating feed-forward loop (31, 32). Type 2 cytokines, especially IL-4 and IL-13, further suppress epidermal differentiation proteins and lipid synthesis, leading to progressive barrier deterioration. This bidirectional interaction between barrier impairment and immune dysregulation represents a fundamental mechanism underlying the persistence and chronicity of AD (**Figure 1**).

**Figure 1:**
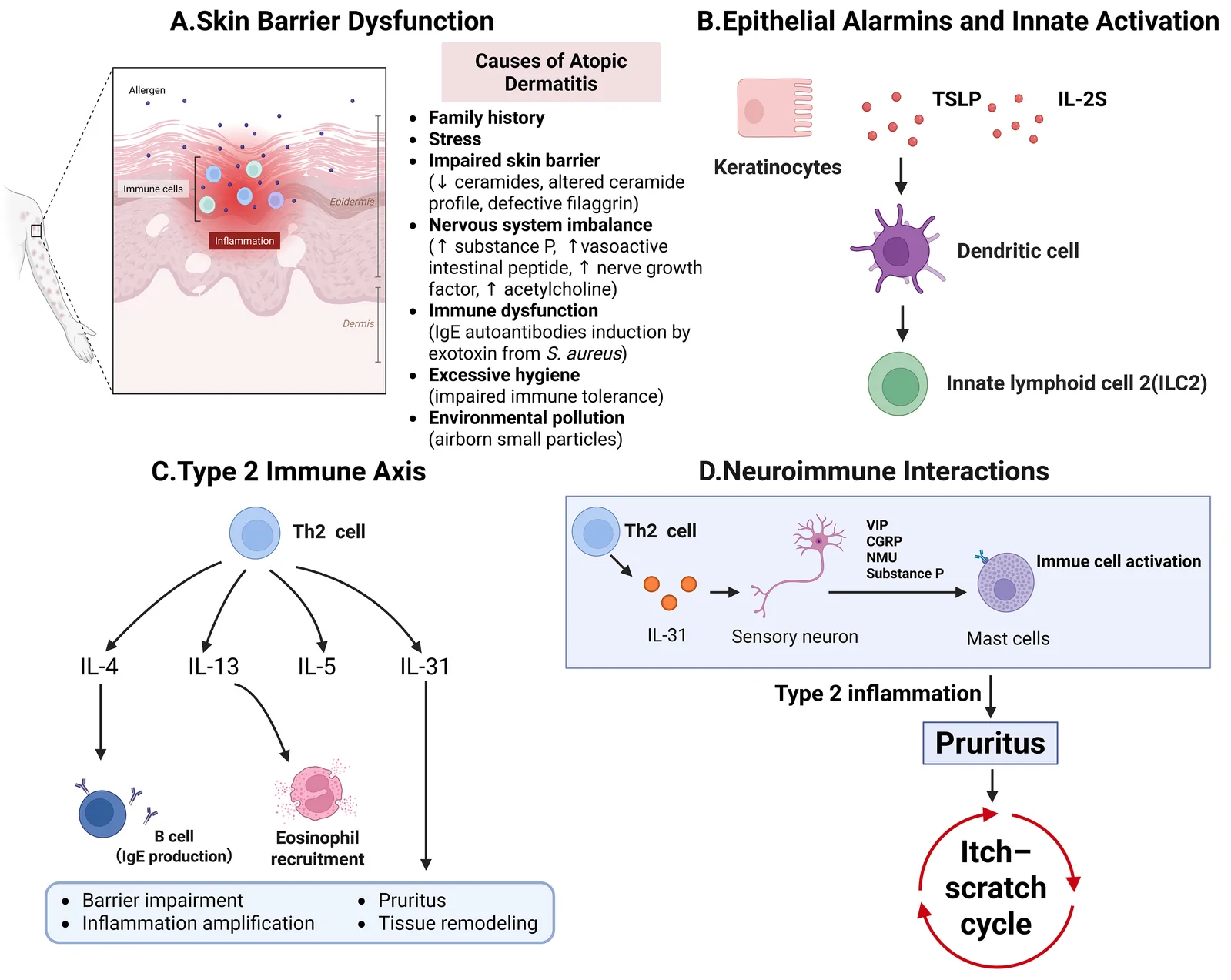
Pathogenesis of atopic dermatitis: from epidermal barrier dysfunction to immune dysregulation and neuroimmune interactions. Epidermal barrier dysfunction initiates the release of epithelial alarmins, leading to activation of innate and adaptive immune responses. Interactions among type 2 immunity, non-type 2 inflammatory pathways, neuroimmune communication, and tissue-resident memory T cells collectively contribute to disease chronicity, recurrence, and therapeutic heterogeneity. These interconnected pathways provide the mechanistic basis for endotype-driven precision medicine and disease-modifying strategies in atopic dermatitis.

### 2.2 Type 2 immunity: a dominant yet incomplete paradigm

Type 2 immunity represents the principal immunological axis in AD and serves as the primary therapeutic target of currently approved biologic agents (27, 29, 33, 34). Following epithelial injury, activated dendritic cells and ILC2s initiate type 2 polarization by promoting the differentiation and expansion of Th2 cells (35). These immune cells subsequently produce a broad spectrum of cytokines, including IL-4, IL-5, IL-13, and IL-31, which collectively coordinate allergic inflammation and tissue remodeling (35).

Although IL-4 and IL-13 share overlapping signaling pathways through the IL-4Rα subunit and downstream JAK–STAT activation, increasing evidence indicates that they exert partially distinct biological functions. IL-4 primarily regulates adaptive immune responses by promoting Th2 differentiation and IgE class switching, whereas IL-13 has more direct effects on epidermal barrier integrity, mucus production, and tissue remodeling (36). This functional divergence may partly explain the differences in clinical responses observed between IL-4Rα blockade and selective IL-13 inhibition.

Type 2 cytokines also directly contribute to pruritus and barrier dysfunction. IL-4 and IL-13 sensitize peripheral sensory neurons and impair epidermal differentiation, whereas IL-31 acts as a potent pruritogenic mediator that directly activates sensory nerve fibers (36). Together, these mechanisms establish a self-amplifying vicious cycle linking inflammation, itch, scratching behavior, and progressive tissue damage (37).

However, the type 2 paradigm alone cannot fully explain the biological complexity of AD. The marked heterogeneity in clinical presentation and therapeutic response suggests that additional immune pathways contribute significantly to disease pathogenesis in specific patient subgroups.

### 2.3 Beyond type 2 inflammation: multiple immune endotypes in AD

Although type 2 inflammation represents the dominant pathogenic pathway in AD, accumulating evidence indicates substantial immunological heterogeneity among patients. Aberrant activation of Th1, Th17, and Th22 pathways has been documented in specific disease phenotypes (38), including intrinsic AD, Asian AD, pediatric AD, and chronic severe disease (20).

Th17-associated cytokines contribute to neutrophilic inflammation and epidermal hyperplasia (39), whereas Th22-derived IL-22 promotes keratinocyte proliferation, epidermal thickening, and tissue remodeling (40). In chronic lesions, enhanced IFN-γ signaling has also been observed and may contribute to disease persistence and the establishment of immunological memory (20). Collectively, these findings support the concept that AD should be regarded as a spectrum of distinct immune endotypes rather than a single, uniform disease entity.

The presence of multiple immune endotypes may partly explain the substantial heterogeneity observed in treatment responses. Patients with predominantly type 2–skewed disease typically exhibit favorable responses to IL-4/IL-13 blockade, whereas individuals with mixed inflammatory profiles may derive greater benefit from broader immunomodulatory approaches, including JAK inhibition (41–45). Accordingly, the identification of endotype-specific biomarkers has become a central goal of precision dermatology and is expected to fundamentally transform future therapeutic decision-making in AD. Recent studies have further advanced this paradigm by identifying candidate endotypes through blood and skin transcriptome profiling (7) and by establishing frameworks that link specific molecular signatures to predicted therapeutic outcomes (18) (**Figure 2**).

**Figure 2:**
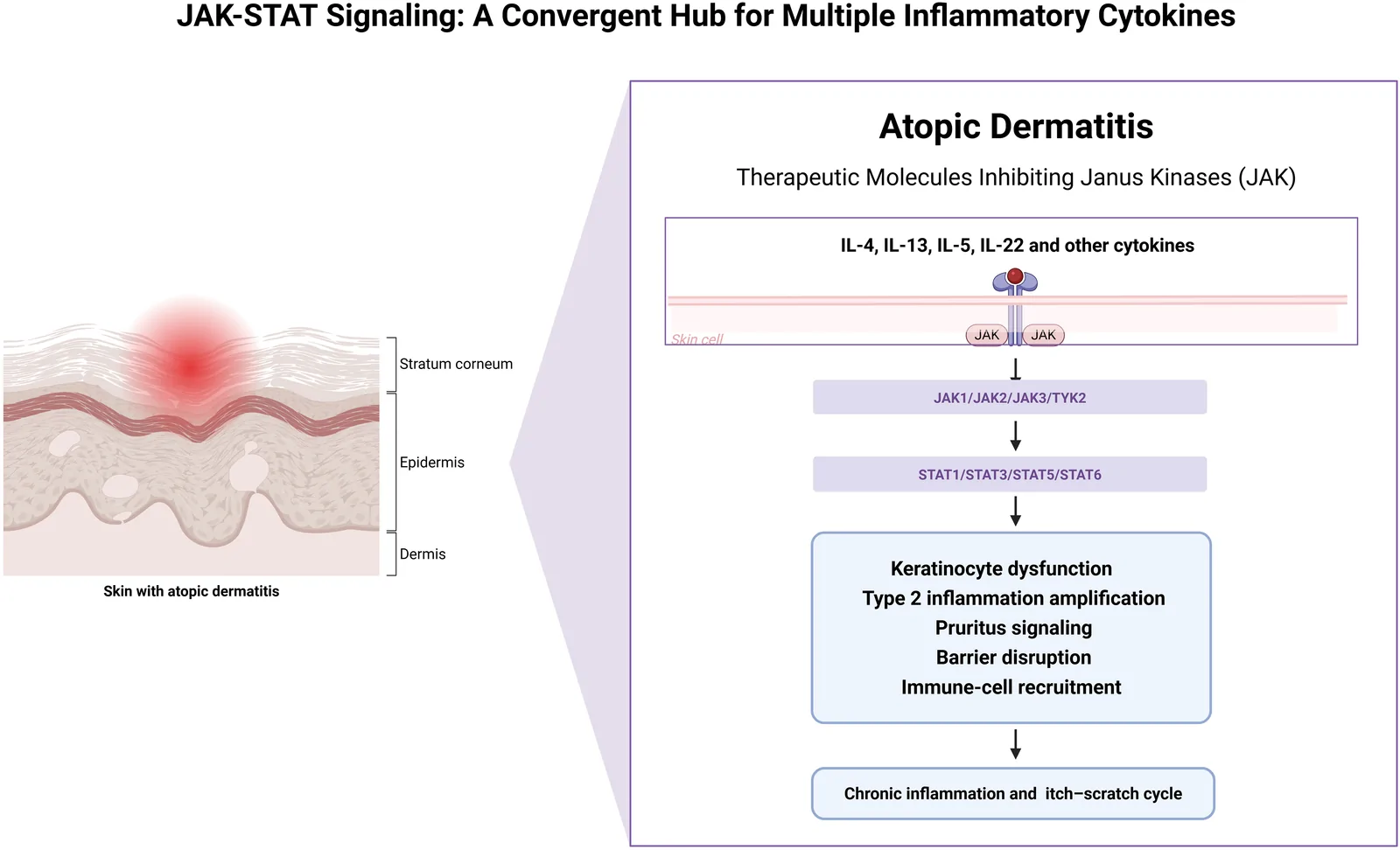
JAK–STAT signaling pathway: a convergent hub of multiple inflammatory cytokines. Cytokines including IL-4, IL-13, IL-31, TSLP, and IFN-γ activate JAK1, JAK2, JAK3, and TYK2 through their respective receptors, leading to phosphorylation and nuclear translocation of STAT1, STAT3, STAT5, and STAT6. This signaling cascade regulates keratinocyte function, inflammatory cell recruitment, pruritus transmission, and epidermal barrier homeostasis. As a central convergence node for multiple inflammatory signals, aberrant activation of the JAK–STAT pathway plays a pivotal role in sustaining chronic inflammation and driving the itch–scratch cycle in atopic dermatitis.

### 2.4 Neuroimmune crosstalk and the itch–scratch cycle

Pruritus is one of the hallmark clinical features of AD and a major determinant of disease burden and impaired quality of life. Traditionally, itch has been considered merely a downstream consequence of cutaneous inflammation. However, accumulating evidence indicates that the nervous system is not a passive recipient of inflammatory signals but actively participates in the initiation and amplification of skin inflammation through bidirectional neuroimmune communication (46).

Type 2 inflammatory cytokines can directly act on peripheral sensory neurons. IL-31, primarily produced by Th2 cells, is a potent pruritogenic cytokine that induces intense itch by activating sensory nerve fibers via the IL-31 receptor complex (47–49). In addition, IL-4 and IL-13 enhance the sensitivity of pruriceptive neurons by modulating neuronal signaling pathways and lowering activation thresholds of sensory nerve fibers. These findings suggest that type 2 cytokines not only regulate immune responses but also directly modulate neuronal function (43, 50).

Conversely, activated sensory neurons actively shape the cutaneous immune microenvironment. They release a range of neuropeptides and neuromodulatory mediators, including substance P, calcitonin gene-related peptide (CGRP), neuromedin U (NMU), and brain-derived neurotrophic factor (BDNF) (51). These neuron-derived factors act on dendritic cells, mast cells, eosinophils, and type 2 innate lymphoid cells (ILC2s), thereby further amplifying type 2 inflammation and promoting tissue remodeling (47–49).

Neuroimmune interactions ultimately establish a self-reinforcing itch–scratch vicious cycle (52–55). Persistent pruritus drives repeated scratching behavior, which in turn further disrupts the epidermal barrier and promotes the release of epithelial alarmins and microbial products (27–29). These events subsequently trigger renewed immune activation and neuronal sensitization, resulting in sustained chronic inflammation and disease progression. Accordingly, a deeper understanding of bidirectional neuroimmune networks provides a strong mechanistic rationale for therapeutic strategies targeting neural pathways, including IL-31 receptor blockade and other emerging neuromodulatory approaches.

### 2.5 Tissue-resident memory T cells and disease recurrence

Despite substantial advances in targeted therapies for AD, disease recurrence remains a major unmet clinical challenge. A considerable proportion of patients achieve clinical remission yet experience rapid relapse after treatment discontinuation, suggesting the presence of persistent pathogenic immune memory within the skin.

Tissue-resident memory T cells (T_RM_) are a subset of memory T cells that reside long-term in peripheral tissues and persist independently of recirculation through the bloodstream. T_RM_ cells typically express surface markers such as CD69 and CD103 and represent a key component of local immune memory, capable of mounting rapid effector responses upon tissue re-exposure to antigenic stimulation (56, 57).

Emerging evidence indicates substantial accumulation of T_RM_ cells in both active lesions and previously affected but clinically resolved skin in AD patients. Upon re-exposure to environmental allergens, microbial antigens, or epidermal injury, these T_RM_ cells can be rapidly reactivated and produce a broad range of inflammatory mediators, including IL-4, IL-13, IL-17, and IL-22, thereby reinitiating inflammatory cascades and promoting disease chronicity and relapse (58).

Notably, T_RM_ cells exhibit marked phenotypic and functional heterogeneity. Distinct T_RM_ subsets may preferentially drive type 2, type 17, or type 22 inflammatory responses (13–15, 20, 39, 59), which may partly explain the substantial inter-individual variability in clinical manifestations and therapeutic responses (60). Moreover, the persistence of pathogenic T_RM_ populations may contribute to suboptimal responses to biologics or rapid relapse following treatment withdrawal in certain patients.

The concept of T_RM_-mediated immune memory is reshaping therapeutic paradigms in AD. Future treatment strategies should extend beyond suppression of acute inflammatory pathways to include immune remodeling, restoration of long-term immune homeostasis, and true disease modification. Accordingly, therapeutic approaches targeting T-cell co-stimulatory pathways, particularly the OX40/OX40L axis, have attracted increasing attention due to their potential disease-modifying effects (20, 61–63).

In summary, current evidence indicates that AD is not merely a type 2 cytokine-driven disorder, but a highly heterogeneous disease involving epidermal barrier dysfunction, multi-pathway immune activation, neuroimmune crosstalk, microbial dysbiosis, and persistent tissue immune memory. These interconnected pathological processes collectively determine disease onset, chronicity, relapse propensity, and therapeutic variability. This integrative framework not only deepens our understanding of AD pathobiology but also provides a strong theoretical foundation for endotype-driven precision medicine and disease-modifying therapeutic strategies.

## 3 Current and emerging targeted therapeutic strategies for atopic dermatitis

With the deepening understanding of the immunopathogenesis of AD, therapeutic strategies have progressively shifted from conventional broad-spectrum immunosuppression toward precision targeting of specific inflammatory pathways. In recent years, the successful application of multiple biologics and small-molecule targeted agents has significantly improved disease control in patients with moderate-to-severe AD. However, substantial inter-individual variability in treatment response persists, with some patients exhibiting primary treatment failure, incomplete efficacy, or rapid relapse after drug discontinuation. These observations further underscore that AD is not driven by a single inflammatory pathway, but rather represents a highly immunologically heterogeneous complex disease. Therefore, a more comprehensive understanding of the mechanisms of action, target populations, and limitations of each therapeutic modality is essential for advancing endotype-driven precision medicine and disease-modifying strategies in AD (**Figure 3**).

**Figure 3:**
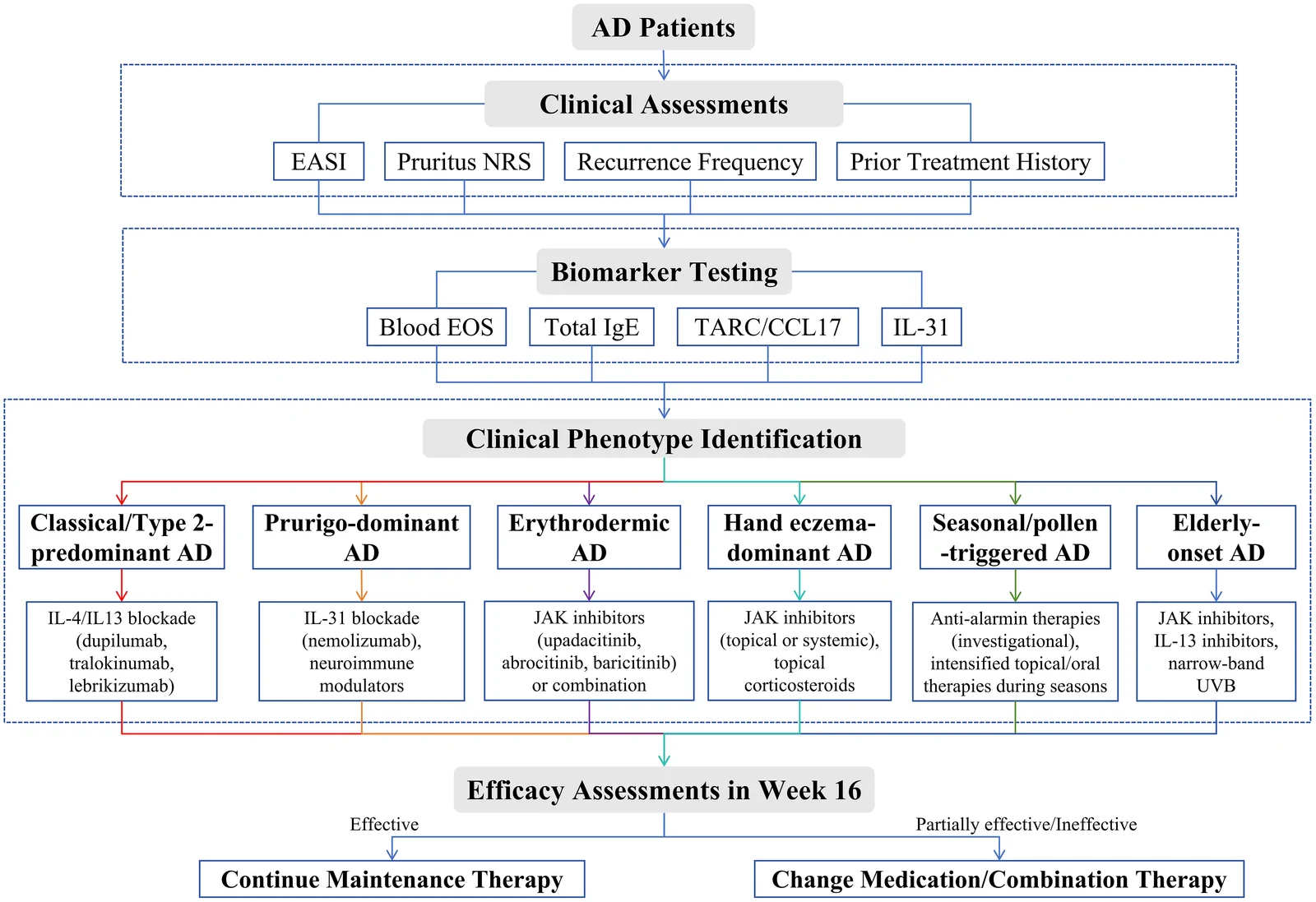
Proposed treatment algorithm for clinical phenotype-based precision therapy in moderate-to-severe atopic dermatitis. This algorithm integrates clinical assessment, biomarker evaluation, clinical phenotype classification, and therapeutic selection to guide individualized treatment decisions. Six clinically recognizable phenotypes are identified based on key clinical features and representative biomarkers as outlined in **Table 2**: classical/type 2-predominant AD, erythrodermic AD, seasonal/pollen-triggered AD, prurigo-dominant AD, hand eczema-dominant AD, and elderly-onset AD. Treatment selection is stratified according to clinical phenotype. Treatment response is evaluated at Week 4 and Week 16 to inform continuation, adjustment, or switching. Patients who achieve sustained remission for ≥ 6 months may be considered for de-escalation, whereas those with relapse should undergo clinical phenotype reassessment. Abbreviations: EASI, Eczema Area and Severity Index; IGA, Investigator's Global Assessment; NRS, numerical rating scale; EOS, eosinophil; TARC, thymus and activation-regulated chemokine; JAKi, Janus kinase inhibitor.

To facilitate comparison among currently available targeted therapies, the mechanisms of action, approved indications, and major clinical characteristics of representative agents are summarized in **Table 1**.

**Table 1:** Current targeted therapies for atopic dermatitis: mechanisms, indications, and clinical characteristics.

Drug	Target	Mechanism of action	Major clinical characteristics	Common adverse events	FDA Approval Status*	Characteristic Biomarker Changes*
Dupilumab (64)	IL-4Rα	Blocks IL-4 and IL-13 signaling	Extensive long-term evidence; improves atopic comorbidities; suitable for long-term maintenance therapy	Injection-site reactions, conjunctivitis	Approved (≥6 months, moderate-to-severe)	Reduces TARC/CCL17, total IgE, and periostin; transient peripheral eosinophilia may occur (65, 66)
Tralokinumab (67)	IL-13	Neutralizes IL-13	Highly selective inhibition of type 2 inflammation; favorable long-term safety profile	Ocular adverse events (conjunctivitis, ocular symptoms) (67), injection-site reactions, upper respiratory infections (69)	Approved (≥12 years, moderate-to-severe)	Prominent reductions in TARC/CCL17 and periostin; may cause transient increases in peripheral eosinophils (70)
Lebrikizumab (71)	IL-13	Inhibits IL-13 signaling by preventing receptor complex formation	Rapid improvement of skin lesions and pruritus; favorable safety profile	Conjunctivitis, injection-site reactions	Approved (≥12 years, moderate-to-severe)	Prominent reductions in TARC/CCL17; may cause transient eosinophilia
Nemolizumab (72)	IL-31RA	Blocks IL-31-mediated itch signaling	Marked antipruritic efficacy; improves sleep disturbance and quality of life	Atopic dermatitis exacerbation/worsening (AD flare) (73), injection-site reactions, nasopharyngitis	Approved (≥12 years, moderate-to-severe, with TCS/TCI)	Reduces serum IL-31-related biomarkers; efficacy may be greater in patients with lower baseline IgE (74)
Upadacitinib (75)	JAK1	Selective JAK1 inhibition	Rapid onset and robust efficacy for skin lesions and pruritus	Acne, upper respiratory infections, herpes zoster, dyslipidemia	Approved (≥12 years, moderate-to-severe)	Suppresses peripheral eosinophil counts; IgE and TARC may not parallel clinical improvement and may remain elevated despite EASI improvement (68)
Abrocitinib (45)	JAK1	Selective JAK1 inhibition	Rapid relief of pruritus and inflammation	Nausea, headache, acne, dyslipidemia	Approved (adults, moderate-to-severe)	Suppresses peripheral eosinophil counts; differential biomarker responses compared to IL-13 inhibitors
Baricitinib (76)	JAK1/JAK2	Dual inhibition of JAK1 and JAK2	Effective in selected patients requiring systemic therapy	Upper respiratory infections, elevated creatine kinase, dyslipidemia	Approved (adults, moderate-to-severe)	Suppresses peripheral eosinophil counts; lipid parameters (HDL, LDL) may increase (77)
Tapinarof (78)	AhR	Activates the aryl hydrocarbon receptor pathway and improves barrier function	Dual effects on barrier repair and inflammation suppression	Folliculitis, contact dermatitis	Approved (≥2 years, mild-to-moderate)	Modulates skin barrier-related biomarkers
Rocatinlimab (79)	OX40	Inhibits OX40-mediated T-cell activation	Phase IIb showed efficacy, but program discontinued (March 2026) due to safety concerns (Kaposi sarcoma); highlights immune surveillance considerations (80)	Upper respiratory infections; malignancy concerns (Kaposi sarcoma) led to program discontinuation (80)	Development discontinued (March 2026)	Biomarker changes under investigation
Amlitelimab (81)	OX40L	Blocks OX40/OX40L co-stimulatory signaling	Potential to induce long-term immune remodeling and sustained remission	Injection-site reactions	Investigational (not FDA-approved)	Biomarker changes under investigation

*FDA approval status as of August 2026. Approved indications may vary among different countries and regions. Please refer to local prescribing information for country-specific approvals.

AD, atopic dermatitis; IL, interleukin; JAK, Janus kinase; PDE4, phosphodiesterase-4; AhR, aryl hydrocarbon receptor; IL-31RA, interleukin-31 receptor A.

### 3.1 Targeting type 2 cytokine signaling: IL-4 and IL-13 blockade

Type 2 inflammation represents the principal pathogenic pathway in AD, with IL-4 and IL-13 regarded as key cytokines driving disease initiation and progression. Currently, biologic agents targeting IL-4 and IL-13 signaling have become a cornerstone of systemic therapy for moderate-to-severe AD.

Although IL-4 and IL-13 share the IL-4 receptor α (IL-4Rα) subunit and converge on the JAK–STAT signaling pathway, increasing evidence suggests that they exhibit partially distinct biological functions (19–21). IL-4 is primarily involved in the initiation of adaptive immune responses, promoting Th2 cell differentiation and B-cell class switching to IgE, thereby driving systemic allergic inflammation. In contrast, IL-13 is more closely associated with local tissue effects, including inhibition of keratinocyte differentiation, disruption of epidermal barrier integrity, promotion of tissue remodeling, and induction of chronic inflammation (82–89).

Dupilumab, the first approved IL-4Rα monoclonal antibody for moderate-to-severe AD, simultaneously blocks both IL-4 and IL-13 signaling and has demonstrated substantial improvements in disease severity, pruritus, and quality of life (21, 86). Subsequently, selective IL-13 inhibitors such as tralokinumab and lebrikizumab have further confirmed the critical role of IL-13 in AD pathogenesis (67, 90).

From a clinical standpoint, the choice among these three agents requires careful consideration of patient-specific characteristics. Dupilumab, as an IL-4Rα antagonist, offers the broadest type 2 blockade and may be particularly advantageous for patients with multiple atopic comorbidities, such as asthma, nasal polyps, or eosinophilic esophagitis, for which it has demonstrated cross-indication efficacy. In contrast, selective IL-13 inhibitors, including tralokinumab and lebrikizumab, provide more refined suppression of the type 2 pathway and may have a slightly more favorable safety profile with respect to conjunctivitis compared to dupilumab.

Lebrikizumab has been associated with rapid onset of efficacy and high rates of skin clearance, making it a compelling option for patients prioritizing early lesion improvement. Tralokinumab has accumulated substantial long-term safety data, supporting its use in patients requiring extended maintenance therapy. Currently, no head-to-head trials have directly compared the three agents, and the selection is often guided by physician experience, patient preference, concomitant allergic conditions, and tolerability considerations. Importantly, for patients who fail to respond adequately to one IL-13 blocker, switching to another agent within the same class is generally not recommended, whereas transition to dupilumab may offer additional efficacy through concurrent IL-4 inhibition. Pooled analysis of five clinical trials demonstrated that conjunctivitis occurred in 7.5% of tralokinumab-treated patients compared with 3.2% in the placebo group; most cases were mild to moderate in severity and resolved during the trial (69).

However, not all patients derive sufficient benefit from IL-4/IL-13 blockade (70, 91). A subset of patients exhibits primary treatment failure, incomplete response, or rapid relapse after treatment discontinuation. Emerging evidence suggests that the coexistence of non–type 2 inflammatory activation, neuroimmune dysregulation, cutaneous microbiome imbalance, and persistent pathogenic tissue-resident memory T cells may collectively contribute to this therapeutic heterogeneity. These findings indicate that the pathogenesis of AD extends beyond type 2 inflammation alone, and that targeting IL-4 and IL-13 signaling may be insufficient to achieve durable disease control in all patients (92).

Therefore, future strategies should incorporate biomarker-based and multi-omics approaches for patient stratification, enabling the identification of individuals most likely to benefit from IL-4/IL-13 blockade and thereby facilitating truly precision-guided therapy.

### 3.2 Targeting pruritus and neuroimmune pathways: IL-31 blockade

Pruritus is one of the hallmark clinical features of AD and a major determinant of impaired quality of life (47–49). Emerging evidence suggests that itch is not merely a consequence of inflammation but also a key driver of disease chronicity.

IL-31 is predominantly produced by Th2 cells and acts directly on sensory neurons through the IL-31 receptor A (IL-31RA) and oncostatin M receptor (OSMR) complex, representing one of the most potent pruritogenic cytokines identified to date (72, 93). IL-31 can activate itch-associated ion channels such as TRPV1 and TRPA1, leading to neuronal sensitization and a reduced threshold for itch perception, thereby promoting persistent pruritus and repetitive scratching behavior (94).

Nemolizumab is a monoclonal antibody targeting IL-31 receptor A. Phase III clinical trials have demonstrated that it rapidly and significantly improves pruritus, while also enhancing sleep quality and overall quality of life. Notably, itch relief with nemolizumab often precedes improvement in skin lesions, further supporting the central role of the neuroimmune axis in AD pathophysiology.

However, IL-31 blockade does not fully suppress cutaneous inflammation, indicating that itch and inflammation, although closely linked, are not entirely dependent on identical biological mechanisms. These findings suggest that pruritus should be considered an independent therapeutic domain in AD and support the concept of a “pruritus-dominant endotype” characterized by severe, treatment-refractory itch.

Therefore, in patients whose primary clinical burden is refractory pruritus and sleep disturbance, IL-31–targeted therapy may represent a particularly valuable precision treatment strategy (72, 93).

Emerging evidence suggests that patient selection may further optimize nemolizumab outcomes. A recent retrospective cohort study demonstrated that patients with intrinsic AD (lacking atopic predisposition) showed significantly better antipruritic responses to nemolizumab compared to those with extrinsic AD, with all 11 intrinsic AD patients achieving a ≥4-point improvement in Peak Pruritus Numerical Rating Scale (PP-NRS4) within 16 weeks, versus only 36% of extrinsic AD patients (74). Furthermore, median total serum IgE levels were significantly lower in responders (74.5 IU/mL) than in non-responders (691.5 IU/mL, p = 0.0034), and multivariate analysis confirmed that higher baseline IgE levels were associated with poorer pruritus outcomes. These findings underscore the importance of baseline IgE levels and atopic predisposition as critical predictors of nemolizumab efficacy in alleviating pruritus, and suggest that nemolizumab may be particularly valuable for patients with intrinsic AD who often have limited treatment options.

### 3.3 Targeting intracellular signaling networks: JAK inhibitors

The JAK–STAT signaling pathway (**Figure 2**) serves as a central hub for transducing signals from multiple inflammatory cytokines and plays a pivotal regulatory role in the pathogenesis of AD (95). A wide range of cytokines, including IL-4, IL-13, IL-31, TSLP, and IFN-γ, exert their biological effects through JAK–STAT–dependent signaling cascades (44, 45).

Unlike biologics that selectively neutralize individual cytokines, JAK inhibitors simultaneously suppress multiple inflammatory pathways, thereby producing broader and more rapid anti-inflammatory effects. Currently approved JAK inhibitors for AD include Abrocitinib, Upadacitinib, and Baricitinib (96–100).

Clinical studies have demonstrated that JAK inhibitors can rapidly alleviate pruritus within days of treatment initiation and significantly reduce disease severity (101). The mechanisms underlying this rapid clinical response may include: (1) concurrent inhibition of multiple type 2 inflammatory pathways such as IL-4, IL-13, and IL-31; (2) direct modulation of sensory neuronal signaling and attenuation of neurogenic sensitization; and (3) simultaneous regulation of non–type 2 inflammatory pathways, including IFN-γ and IL-22 (43, 50). Accordingly, JAK inhibitors are particularly suitable for patients with highly active inflammation, severe pruritus, or mixed immunological phenotypes.

When selecting among Upadacitinib, Abrocitinib, and Baricitinib, clinicians should consider distinctions in their pharmacological profiles, efficacy strength, and safety signals. Upadacitinib and Abrocitinib are highly selective JAK1 inhibitors, whereas Baricitinib also inhibits JAK2, which may contribute to distinct laboratory effects, including changes in hematologic parameters and creatine kinase levels. Head-to-head data suggest that Upadacitinib may offer superior efficacy in achieving skin clearance (EASI 90), particularly in patients with severe or treatment-refractory disease, although this benefit must be balanced against a slightly higher incidence of acne, herpes zoster, and dose-dependent laboratory abnormalities. Abrocitinib has shown comparable efficacy with a distinct adverse event profile, notably dose-related nausea and headache, which are often transient and managed with dose titration or concomitant food intake. Baricitinib, while somewhat less potent in skin clearance compared to the selective JAK1 inhibitors, remains a valuable option for patients with moderate disease or those who have contraindications to other systemic therapies. Age, renal function, cardiovascular risk factors, and history of thromboembolic events are crucial considerations for all JAK inhibitors, consistent with class safety warnings. For patients with inadequately controlled type 2 inflammation despite biologic therapy, or those presenting with mixed inflammatory features, JAK inhibitors offer an effective rescue or alternative strategy, but individualized risk-benefit assessment remains paramount.

Importantly, the biomarker changes observed with JAK inhibitors differ fundamentally from those seen with biologics. While IL-4/IL-13 blockade reduces TARC/CCL17 and IgE in parallel with clinical improvement, JAK inhibition characteristically suppresses peripheral eosinophil counts but may not produce corresponding reductions in IgE or TARC. Indeed, IgE and TARC may remain elevated or even increase at later time points despite improvement in EASI or pruritus (68). This dissociation between clinical response and conventional type 2 biomarkers during JAK inhibition highlights the need for mechanism-specific monitoring strategies and suggests that different biomarkers may be required to track treatment response depending on the therapeutic class. A direct real-world comparison has demonstrated the differential association of eosinophil reduction with JAK1 inhibitor response and TARC reduction with IL-13 antibody response (102), further supporting the concept that biomarker changes are pathway-specific rather than simply reflecting disease improvement.

The rapid efficacy of JAK inhibition further highlights the central role of intracellular signaling convergence in AD pathogenesis and, from another perspective, supports the concept of multiple immune endotypes beyond classical type 2 inflammation.

However, given their broad mechanism of action, long-term use of JAK inhibitors requires careful consideration of potential safety risks, including infections, herpes zoster, thromboembolic events, and laboratory abnormalities (103). Therefore, balancing efficacy and long-term safety remains an important area for future investigation.

For patients with localized or mild-to-moderate disease, topical JAK inhibitors (e.g., ruxolitinib cream) provide an important therapeutic bridge between topical corticosteroids and systemic therapies (78, 104, 105). Current standard topical treatments for AD include topical corticosteroids, calcineurin inhibitors, and the phosphodiesterase-4 inhibitor crisaborole (106). These agents effectively avoid corticosteroid-induced skin atrophy while providing robust control of local inflammation and supporting barrier restoration.

At the same time, the broader immunomodulatory effects of systemic JAK inhibition may be associated with increased risks of infections, herpes zoster, and thromboembolic events with long-term use (107–109).

### 3.4 Targeting epithelial alarmins: TSLP and IL-33 blockade

Epithelial alarmins represent the most upstream mediators in the inflammatory cascade of AD and serve as critical bridges linking epidermal barrier disruption to type 2 immune activation (110). TSLP, IL-25, and IL-33 can activate dendritic cells and type 2 innate lymphoid cells (ILC2s), thereby promoting Th2 polarization and amplifying type 2 inflammation (27–29). Accordingly, targeting alarmins has long been considered a promising strategy with potential disease-modifying effects.

However, clinical outcomes from trials targeting TSLP and IL-33 have not fully met initial expectations. Although preclinical studies in animal models demonstrated robust anti-inflammatory effects following alarmin blockade, clinical trials in humans have shown only modest or limited efficacy in subsets of patients.

This discrepancy between preclinical and clinical findings suggests that AD may not be driven by a single unified upstream mechanism. Instead, different patient subgroups may exhibit distinct immune initiation pathways, and alarmin dependency may be restricted to specific disease endotypes.

Therefore, future efforts should integrate biomarkers, multi-omics approaches, and machine learning–based stratification to identify patient populations most likely to benefit from alarmin-targeted therapies, thereby advancing endotype-driven precision intervention in AD.

### 3.5 Targeting immune memory and costimulatory pathways: OX40/OX40L

Although current targeted therapies can effectively suppress active inflammation, most patients still fail to achieve sustained remission, and disease relapse after treatment discontinuation remains common. These observations suggest that the therapeutic goal in AD should extend beyond inflammation control to modification of the natural disease course.

OX40 is a costimulatory molecule expressed on activated T cells, whereas OX40L is primarily expressed on dendritic cells and other antigen-presenting cells (111–113). The OX40/OX40L signaling axis promotes T-cell activation, proliferation, survival, and memory formation, and may also contribute to the maintenance of pathogenic tissue-resident memory T cells (91, 114).

Increasing evidence suggests that blockade of the OX40/OX40L pathway can induce durable clinical improvement and maintain a degree of disease remission even after treatment discontinuation (115, 116). These findings indicate that OX40/OX40L inhibition may not only suppress acute inflammation but also achieve disease-modifying effects through modulation of pathogenic immune memory (116).

The OX40/OX40L axis has emerged as an important therapeutic target for disease modification in AD. Telazorlimab, Rocatinlimab, and Amlitelimab have all demonstrated promising clinical efficacy in trials. Telazorlimab is an anti-OX40 IgG1 monoclonal antibody. In a phase IIb randomized, placebo-controlled study, patients receiving 300 mg or 600 mg subcutaneous injections for 16 weeks showed significantly greater improvements in Eczema Area and Severity Index (EASI) scores compared with placebo, with an overall favorable safety profile. The most common adverse events included exacerbation of AD, nasopharyngitis, upper respiratory tract infections, viral infections, and headache, with only one patient discontinuing treatment due to drug-related paresthesia.

Rocatinlimab is another anti-OX40 monoclonal antibody. Phase IIb data demonstrated that all dose groups achieved significantly greater reductions in EASI scores compared with placebo at week 16, with responses continuing to improve and maintained up to week 56. Improvements in sleep quality and quality of life were also observed, and most clinical benefits persisted for approximately 20 weeks after treatment cessation (91). The most common adverse events included pyrexia, nasopharyngitis, and chills.

Amlitelimab is a monoclonal antibody targeting OX40L (116). A phase IIa randomized, placebo-controlled trial demonstrated that even low-dose (200 mg) intravenous administration significantly improved EASI scores. Reported adverse events included headache, hyperhidrosis, upper respiratory tract infection, pyrexia, elevated aspartate aminotransferase levels, and iron-deficiency anemia, with some patients discontinuing treatment due to AD exacerbation or nasopharyngitis.

Overall, OX40/OX40L blockade not only significantly improves clinical manifestations of AD but may also provide more sustained disease control after treatment cessation. This suggests that these agents may achieve immune remodeling and disease modification by interfering with T-cell activation and the formation and maintenance of tissue-resident memory T cells, making them conceptually promising candidates for long-term remission and relapse prevention.

Although this hypothesis requires confirmation through long-term follow-up studies, therapeutic strategies targeting immune memory remodeling are reshaping the treatment goals of AD, shifting the focus from short-term symptom control toward long-term immune homeostasis restoration and true disease modification. However, the discontinuation of the rocatinlimab program due to safety concerns serves as a critical reminder that the clinical development of immunomodulatory approaches targeting T-cell memory must carefully balance efficacy against potential risks to immune surveillance.

Collectively, targeted therapies in AD have evolved from simple cytokine blockade to a multilayered precision intervention system targeting cytokine networks, neuroimmune axes, and immune memory. Future therapeutic strategies will likely move beyond symptom control toward endotype-driven precision medicine and disease-modifying approaches.

The marked heterogeneity in therapeutic responses among patients with atopic dermatitis suggests the existence of distinct immunological endotypes. Increasing evidence supports the concept that clinical phenotype-driven stratification, integrated with biomarker assessment, may facilitate individualized therapeutic selection and ultimately enable precision medicine in AD. A clinically oriented framework that first reflects recognizable AD phenotypes—such as classical/type 2-predominant, erythrodermic, seasonal/pollen-triggered, prurigo-dominant, hand eczema-dominant, and elderly-onset AD—and subsequently integrates their immunological characteristics and biomarkers is proposed in **Table 2**.

**Table 2:** Clinical phenotype-based framework for precision therapeutic selection in atopic dermatitis.

Clinical phenotype	Key clinical features	Immunological characteristics	Representative biomarkers	Potential therapeutic approaches
Classical/Type 2-predominant AD	Early-onset, flexural eczema, elevated IgE, personal/family history of atopy	Th2-skewed with elevated IL-4, IL-13, eosinophilic inflammation	Elevated total IgE, blood eosinophilia, TARC/CCL17, periostin	IL-4/IL-13 blockade (dupilumab, tralokinumab, lebrikizumab)
Erythrodermic AD	Extensive (>80% BSA), severe erythema and scaling, often refractory to topical therapies	Severe type 2 with possible Th17/Th22 involvement	Highly elevated IgE, TARC, LDH	JAK inhibitors (upadacitinib, abrocitinib, baricitinib) or combination approaches
Seasonal/pollen-triggered AD	Fluctuating disease activity with seasonal exacerbations (spring/autumn), history of pollinosis	Epithelial alarmin-driven with seasonal immune activation	TSLP, IL-33, seasonal IgE spikes	Anti-alarmin therapies (investigational), intensified topical/oral therapies during seasons
Prurigo-dominant AD	Prominent prurigo nodules, severe intractable pruritus, sleep disturbance, often disproportionate to erythema severity	Neuroimmune axis activation with prominent IL-31 signaling	Elevated serum IL-31, high itch NRS score	IL-31 blockade (nemolizumab), neuroimmune modulators
Hand eczema-dominant AD	Predominant or exclusive hand/foot involvement, often occupational aggravation	Mixed type 2 and type 1/17 inflammation	Variable IgE, often lower eosinophilia	JAK inhibitors (topical or systemic), topical corticosteroids
Elderly-onset AD	First onset after age 60, often male predominance, less pronounced erythema, lower IgE levels	Often lower IgE, Th17/Th22 involvement, distinct microbiome profile	Lower IgE, elevated IL-17/IL-22	JAK inhibitors, IL-13 inhibitors, narrow-band UVB

This framework emphasizes that clinical phenotypes should be the primary consideration in therapeutic decision-making, with immunological characteristics and biomarkers serving as supplementary information to refine treatment selection. The phenotypes listed above are not mutually exclusive, and patients may present with overlapping features. Treatment selection should be individualized based on the predominant clinical pattern, disease severity, patient preferences, and treatment availability.

## 4 Clinical challenges, unmet needs, and future perspectives

Despite substantial advances in biologics and small-molecule inhibitors in recent years that have significantly improved outcomes in AD, achieving long-term sustained remission remains a central challenge in clinical practice. Although current therapies effectively reduce disease activity and improve quality of life, a considerable proportion of patients still experience incomplete responses, secondary treatment failure, or rapid relapse after discontinuation. These findings suggest that suppression of active inflammation alone may be insufficient to fundamentally alter the natural course of AD.

One of the key determinants limiting durable disease control is the marked heterogeneity of AD. Increasing evidence indicates that AD is not a single disease entity but rather a complex spectrum composed of multiple immune endotypes, including a type 2–high endotype dominated by type 2 inflammation, a pruritus-dominant endotype characterized by neuroimmune dysregulation, a mixed hyperinflammatory endotype involving activation of Th1/Th17/Th22 pathways, and a relapse-associated endotype driven by persistent T_RM_. This biological heterogeneity helps explain why patients with similar clinical phenotypes may exhibit markedly different therapeutic responses. However, current clinical practice still relies primarily on disease severity rather than immunological mechanisms to guide treatment decisions, resulting in a persistent disconnect between pathobiology and clinical management (117).

This issue is further compounded by the lack of robust biomarkers in precision medicine. Although serum IgE levels, peripheral blood eosinophil counts, and TARC/CCL17 can partially reflect disease activity, their ability to predict therapeutic response, disease progression, and long-term outcomes remains limited. Current research on AD biomarkers has identified several promising candidates: TARC/CCL17 has emerged as one of the most reliable indicators of AD disease activity, correlating strongly with SCORAD and EASI scores (118); periostin, IL-13, and IL-22 also show consistent utility in grading severity and correlating with treatment outcomes; and SCCA2 has been shown to outperform IgE as a reliable severity biomarker (118). Furthermore, type 2 markers have been demonstrated to decrease with dupilumab and tralokinumab treatment, matching clinical gains (118). However, conventional biomarkers including total IgE and peripheral eosinophil count show less clinical utility than these newer biomarkers (118). Given the highly dynamic and multidimensional nature of AD immunopathology, single biomarkers are insufficient to fully capture disease complexity. Future research is therefore likely to rely on multi-omics integration and computational modeling to identify biologically meaningful endotypes and enable individualized treatment strategies.

Among unresolved mechanisms, disease recurrence remains one of the most challenging clinical problems. Increasing evidence indicates that clinical remission does not equate to immunological remission. T_RM_ can persist long-term in previously affected skin, sustained by local cytokines such as IL-7, IL-15, and TGF-β. Upon re-exposure to environmental triggers, T_RM_ cells can be rapidly reactivated and produce cytokines including IL-4, IL-13, IL-17, and IL-22, thereby reinitiating keratinocyte activation, barrier disruption, and inflammatory cascades. This persistent immune memory provides a mechanistic explanation for the chronic relapsing nature of AD and the limitations of current therapies in achieving durable remission.

In summary, these challenges collectively highlight that future AD management must shift from simple inflammation suppression toward restoration of cutaneous immune homeostasis. This transition integrates disease heterogeneity, biomarker development, and immune memory mechanisms into a unified conceptual framework. Accordingly, future therapeutic paradigms are expected to evolve along three directions: (1) from phenotype-based treatment to endotype-driven precision medicine; (2) from single-pathway inhibition to multi-layer immune network modulation; and (3) from disease control to immune remodeling (119).

Ultimately, disease modification in AD will not only imply sustained symptom control but also long-term restoration of immune homeostasis across barrier, immune, neural, and memory systems (120). Achieving this goal will require early intervention, biomarker-guided stratification, and the capacity to reprogram pathogenic immune memory. From this perspective, AD is increasingly being redefined as a chronic ecosystem disease, providing a new conceptual foundation for precision dermatology and the era of immune remodeling.

Beyond biological and clinical parameters, patient attitudes and preferences play an increasingly critical role in the selection of systemic therapies for AD. When choosing among available targeted agents, multiple patient-centered factors must be carefully considered: age (pediatric, adult, or elderly) influences both drug eligibility and tolerability; disease severity and the intensity of pruritus guide the choice between biologics and JAK inhibitors, with the latter offering more rapid itch relief; patient expectations regarding onset of action, route of administration (subcutaneous injection vs. oral), and frequency of dosing significantly affect treatment adherence and satisfaction. Furthermore, fears of adverse effects—particularly infections, thromboembolic events, and laboratory abnormalities with JAK inhibitors, or injection-site reactions and conjunctivitis with biologics—must be addressed through thorough patient education and shared decision-making. Pregnancy planning and contraceptive use are crucial considerations for women of childbearing age, as safety data for most targeted therapies in pregnancy remain limited. Cost, insurance coverage, and drug accessibility also substantially influence treatment choices across different healthcare systems. Ultimately, the ability and willingness to adhere to long-term treatment regimens, combined with realistic expectations about achievable outcomes, should be integrated into a holistic, patient-centered therapeutic strategy that respects individual values and lifestyle factors.

## 5 Conclusion

AD is a highly heterogeneous and dynamically evolving chronic inflammatory skin disease involving complex interactions among epidermal barrier dysfunction, immune dysregulation, neuroimmune crosstalk, cutaneous microbial imbalance, and persistent tissue immune memory. Increasing evidence indicates that AD is no longer a purely type 2 inflammatory disorder but should instead be conceptualized as an integrated cutaneous ecosystem disorder composed of multiple pathogenic endotypes.

With the deepening understanding of AD immunopathogenesis, the development of novel targeted therapies—including agents directed against IL-4/IL-13, IL-31, the JAK signaling pathway, epithelial alarmins, and T-cell costimulatory axes—has significantly improved disease control and patient outcomes. However, challenges such as treatment response heterogeneity, lack of reliable biomarkers, disease relapse after treatment discontinuation, and difficulty in achieving durable remission remain major unmet clinical needs.

Future therapeutic strategies are expected to shift from empirical treatment toward biomarker-guided, endotype-based stratification integrated with multi-omics technologies to enable individualized therapeutic decision-making. More importantly, treatment goals will progressively evolve from short-term inflammation control toward long-term immune remodeling and true disease modification. Further elucidation of disease heterogeneity and the biological basis of immune memory formation is anticipated to facilitate the development of more precise, durable, and disease-modifying strategies, ultimately improving long-term remission rates and quality of life in patients with AD.

Collectively, the evolving paradigm of AD management is shifting from phenotype-based disease control toward endotype-driven precision medicine and ultimately toward disease modification and durable remission.
